# Antioxidant dietary patterns mitigate pain and raise psychological wellbeing in ovarian endometrioma: surgical status shapes associations

**DOI:** 10.3389/fnut.2026.1921186

**Published:** 2026-08-12

**Authors:** Emanuela Spagnolo, María Rey Hernández, Blanca Díaz Fuentes, Carolina Fernández-Cuesta Morín, Ana López, Carmen Céspedes Casas, Javier García-Rodríguez, Alicia Hernández, David Ramiro-Cortijo

**Affiliations:** 1 Department of Obstetrics and Gynecology, Faculty of Medicine, Universidad Autónoma de Madrid, Madrid, Spain; 2 Endometriosis Unit, Hospital Universitario La Paz, Madrid, Spain; 3 Hospital La Paz Institute for Health Research (IdiPAZ), Hospital Universitario La Paz, Madrid, Spain; 4 Department of Physiology, Faculty of Medicine, Universidad Autónoma de Madrid, Madrid, Spain; 5 Instituto Universitario de Estudios de la Mujer (IUEM), Universidad Autónoma de Madrid, Madrid, Spain

**Keywords:** antioxidant dietary pattern, gynecological function, inflammatory dietary pattern, ovarian endometrioma (OMA), pain perception, psychological wellbeing, quality of life

## Abstract

**Introduction:**

Endometriosis is a systemic inflammatory disease in which immune dysregulation and oxidative stress contribute to pain and impaired quality of life. This study aimed to examine whether antioxidant and inflammatory dietary patterns are associated with pain, sexual and reproductive function, and psychological wellbeing in women with ovarian endometrioma (OMA), and whether these associations differ according to surgical status.

**Methods:**

This observational cross-sectional study included women with OMA undergoing hormonal therapy, classified according to whether they had surgery (S, *n* = 10) or not (NS, *n* = 17), and healthy controls (*n* = 15). Dietary intake was assessed using a validated food frequency q uestionnaire to derive antioxidant and inflammatory dietary patterns, while pain, sexual and reproductive function, and psychological wellbeing were evaluated using standardized questionnaires, together with systemic immune and biochemical markers. Multivariate and principal component analyses were used to examine associations between dietary patterns (Nut PCs), immune markers, and functional outcomes.

**Results:**

Women with OMA showed significantly lower psychological wellbeing, sexual and reproductive function, and higher pain sensitivity compared with controls, with poorer reproductive function in OMA NS. Lymphocyte levels were higher in women with OMA. Higher antioxidant dietary scores (Nut PC1 and DAI) were associated with lower pain sensitivity and better psychological wellbeing. In contrast, protein-related patterns (Nut PC2) were positively, and lipid-related patterns (Nut PC3) were negatively associated with menstrual characteristics and sexual function. Psychological wellbeing was inversely associated with OMA and but positively related to antioxidant dietary patterns. Antioxidant dietary index (DAI) was higher in women with OMA S than in controls and OMA NS, while dietary inflammatory index (DII) was lower in OMA NS versus control, with no differences compared to OMA S.

**Discussion:**

Nutrition may modulate symptom perception in women with OMA undergoing hormonal therapy, particularly through antioxidant-rich dietary patterns. These associations appear to be influenced by surgical history and systemic inflammation. Integrating personalized nutritional counseling alongside conventional treatments may offer a feasible strategy to enhance quality of life in this population.

## Graphical abstract

**Figure:**
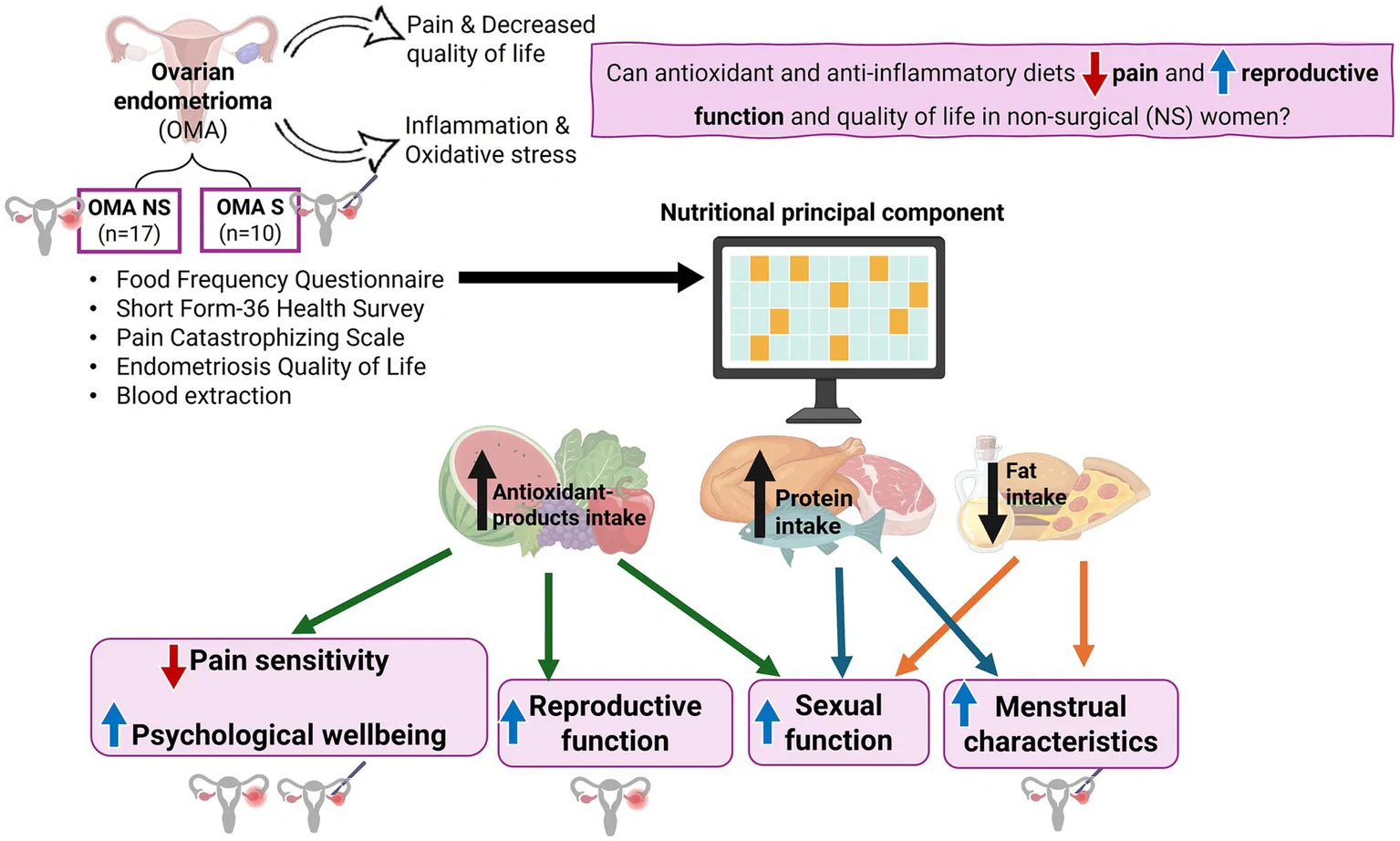

This study investigated whether antioxidant and anti-inflammatory dietary patterns are associated with pain perception, reproductive and sexual function, and psychological wellbeing in women with ovarian endometrioma (OMA), and whether these relationships differ according to surgical status. Women with OMA were classified as non-surgical (NS) or surgical (S) and compared with healthy controls. Higher adherence to antioxidant-rich dietary patterns was associated with lower pain sensitivity and improved psychological wellbeing, as well as better reproductive outcomes. In contrast, protein- and lipid-related dietary patterns showed differential associations with menstrual characteristics and sexual function.

## 1 Introduction

Endometriosis, an estrogen-dependent disorder, is characterized by the presence of endometrial-like tissue outside the uterine cavity, affecting approximately 10% of reproductive age women. In addition, endometriosis is recognized as a systemic inflammatory disease, involving immune dysregulation, oxidative stress, and altered neuroendocrine signaling, which together contribute to pain, reproductive dysfunction, and impaired quality of life (1). Ovarian endometrioma (OMA), one of the most prevalent phenotypes, is associated with chronic pelvic pain, sexual dysfunction, reduced vitality, and psychological distress, even among women receiving hormonal treatment.

Accumulating evidence suggests that immune activation and redox imbalance play a central role in endometriosis pathophysiology. Elevated circulating inflammatory markers, including lymphocytes and pro-inflammatory cytokines, have been linked to pain severity, and mental distress outcomes in this pathological condition (2). These immune mediators can influence nociceptive pathways, hypothalamic–pituitary–adrenal axis regulation, and neurotransmitter metabolism, connecting inflammation with psychological wellbeing and symptom perception (3, 4). As a result, modifiable lifestyle factors have gained attention as potential adjunctive therapeutic targets. Among these factors, diet has emerged as a key modulator of inflammation and oxidative status. Observational studies indicate that unhealthy dietary habits are associated with pain, sexual dysfunction, and decrease quality of life in women with OMA (5). Conversely, dietary patterns rich in antioxidants, fiber, and unsaturated fatty acids may exert immunomodulatory effects in endometriosis, reducing oxidative stress and modulating innate immune responses (6–8).

Despite growing interest, interventional evidence remains limited. Randomized clinical trials (RCTs) evaluating anti-inflammatory and antioxidant dietary interventions in endometriosis suggest modest, but promising benefits, reducing pelvic pain, oxidative stress biomarkers, gastrointestinal symptoms, and improvements in dyspareunia and dyschezia (9–11). However, these RCTs are constrained by small sample sizes, short intervention durations, and heterogeneous outcome measures. Consequently, whether dietary modulation can influence pain, sexual function, and psychological wellbeing, particularly in women receiving hormonal therapy, remains insufficiently understood.

The management of OMA is primarily based on long-term hormonal therapy, particularly in young women with reproductive desire, with the aim of controlling symptoms and preventing disease progression (12–14). Within this framework, additional strategies that may improve symptom burden and quality of life are of increasing interest. Previous findings showed that women with OMA who did not undergo surgical treatment exhibited poorer sexual function and occupational performance compared with surgically treated women (15), highlighting the clinical heterogeneity of this population when quality-of-life outcomes are assessed. In this context, diet should be considered a complementary approach rather than a primary treatment, with potential to modulate inflammation, pain perception, and psychosocial wellbeing. However, its role in women with OMA remains insufficiently explored.

Based on this background, it was hypothesized that adherence to antioxidant-rich and anti-inflammatory dietary patterns in women with OMA undergoing hormonal treatment may be associated with improved pain perception, sexual and reproductive function, and psychological wellbeing, potentially exerting effects comparable to those observed after surgical intervention. Thus, the aim of this study was to assess whether antioxidant and inflammatory dietary patterns are differentially associated with functional and wellbeing outcomes according to surgical status, and whether systemic immune markers may contribute to these relationships.

## 2 Materials and methods

### 2.1 Study design and participants

This observational, cross-sectional study included women diagnosed with OMA. Participants with OMA were recruited from the Endometriosis Unit of the Gynecology Service at Hospital Universitario La Paz (HULP, Madrid, Spain). OMA was diagnosed based on clinical assessment and imaging, in accordance with current guidelines (16). Transvaginal ultrasound performed by experienced clinicians was the primary diagnostic tool, identifying typical endometrioma features such as a homogeneous “ground-glass” ovarian cyst, with magnetic resonance imaging used when ultrasound findings were inconclusive. Eligible participants were premenopausal women aged between 25 and 45 years with a confirmed diagnosis of OMA. Inclusion criteria comprised the absence of a previous diagnosis of cancer, emotional or psychiatric disorders, hypertension, or obesity, and the absence of ongoing hormone therapy unrelated to endometriosis management. Exclusion criteria included drug abuse and a diagnosis of deep endometriosis.

All women in the OMA group were receiving a continuous regimen of hormonal treatment for endometriosis [progestins and combined oral contraceptives have been shown to improve pain symptoms (17)] with the absence of menstrual bleeding during the assessment period. OMA group was classified according to surgical status: women who had undergone surgery due to endometriosis-related symptoms (OMA S) and women without surgery (OMA NS). Control participants were recruited from women attending routine gynecological follow-up visits at HULP. Eligible controls had no previous diagnosis of endometriosis nor history of gynecological surgery. To reduce the likelihood of undiagnosed endometriosis, all control participants underwent detailed gynecological examination and high-resolution transvaginal ultrasound.

Participant recruitment took place between October 2024 and December 2025. In total, 42 women provided written informed consent and were included in the study (control, *n* = 15; OMA NS, *n* = 17; OMA S, *n* = 10). Given the exploratory nature of the study, no formal sample size calculation was performed. However, to provide an estimate of the statistical sensitivity of the sample, an effect size analysis was conducted using G*Power version 3.1.9.7 (Heinrich Heine University Düsseldorf, Germany). As G*Power does not implement a specific procedure for the non-parametric test, the equivalent one-way fixed-effects ANOVA model with independent groups was used. Assuming a two-sided significance level of 5% and a statistical power of 80%, the available sample of 42 participants was sufficient to detect a large effect size (Cohen’s *f* = 0.498).

The duration of OMA progression ranged from 1.0 to 5.8 years. All women in the OMA groups presented with ovarian cysts, which were bilateral in 23.1% of women in the OMA NS group and in 71.4% of women in the OMA S group. The most frequently prescribed hormonal treatment in the OMA NS group was progestogen-based therapy (46.2%), whereas combined hormonal therapy was more prevalent among women in the OMA S group (57.1%). Regarding symptomatology, dysmenorrhea was the most incapacitating symptom reported by women in the OMA NS group (76.9%), followed by dyspareunia (60.0%). In contrast, abnormal uterine bleeding was the most reported debilitating symptom among women in the OMA S group (28.6%).

Participants completed a sociodemographic questionnaire including age, nationality, educational level, monthly income, smoking habits, special diet use (e.g., gluten-free or vegetarian) and information on dietary supplements was used. In addition, the food frequency questionnaire (FFQ) and perceived pain, functional and wellbeing outcomes were collected. The women were scheduled to take an 8 mL blood sample by venipuncture. This sample was used to determine blood cells (neutrophils, lymphocytes, monocytes, eosinophils, basophils and platelets) and biochemical parameters. Biochemical parameters (prothrombin, glucose, total cholesterol, HDL-cholesterol, LDL-cholesterol, triglycerides, urea, and creatinine) were measured to characterize the participants’ metabolic status. Additional biomarkers were assessed to evaluate systemic inflammation (C-reactive protein, fibrinogen, ferritin, and cortisol) and antioxidant and immunomodulatory status (vitamin D, bilirubin, and albumin). These parameters provided a comprehensive assessment of systemic factors that may act as potential confounders when interpreting the findings. These samples were processed by clinical medicine laboratory of HULP. Additionally, weight (kg) was extracted from medical records.

Compliance with ethical standards. This design has the approval of the Research Ethical Committee of HULP (PI-5435; approved on 02 December 2022).

### 2.2 Nutritional and dietary pattern

Dietary intake was assessed using the Spanish version of the FFQ (18–20), a validated tool designed to estimate habitual food consumption over a specific period (21, 22). FFQ estimate food groups and nutrient intakes and classify women by dietary patterns. FFQ included 137 food items with specified portion sizes and offer 9 frequency response options. The collected data was processed and analyzed using the Food Frequency Questionnaire European Transformation Algorithm (23), an open source that processes dietary data from the FFQ. This tool has been validated for use in Spanish adults (24, 25). The monitoring, completion of the FFQ, and its correction were coordinated by a specialist nutritionist. More details are shown in Spagnolo et al. (5). In the present study was extracted nutritional elements related to inflammatory diets and antioxidants related to endometriosis: antioxidant vitamins, such as vitamin C (ascorbic acid, mg/day), vitamin E (*α*-tocopherol, mg/day), vitamin A (retinol equivalents, μg/day); antioxidant nutrients, such as *β*-carotene (μg/day), total carotene (μg/day) and folate (μg/day); immunomodulatory minerals, such as selenium (μg/day), zinc (mg/day), magnesium (mg/day) and iron (mg/day) (26); dietary fiber (non-starch polysaccharides, g/day) (27), fats (g/day), cholesterol (mg/day), and fatty acids, such as saturated fatty acids (SFAs, g/day); monounsaturated fatty acids (MUFAs, g/day); polyunsaturated fatty acids (PUFAs, g/day), vitamin D (μg/day) (28); carbohydrates (g/day), total sugars (g/day) and refined sugars, such as sucrose (g/day); proteins (g/day) and processed meats, and sodium (mg/day) (29). In addition, nutritional categories in g/day such as fruits, vegetables, meat products, fish products, and snacks were extracted. All nutritional elements were standardized per 1,000 kcal/day of energy intake. In addition, the dietary antioxidant index and dietary inflammatory index were calculated.

The Dietary Antioxidant Index-DAI (30) was computed to quantify adherence to a dietary antioxidant pattern based on the intake of key antioxidant nutrients, including vitamin A, vitamin C, vitamin E, zinc, selenium and magnesium. For each nutrient, standardized scores were calculated using the formula (individual intake − global mean) / global standard deviation, and the DAI was derived by summing these standardized values. Higher DAI scores reflect greater overall antioxidant intake and have been associated with a more favorable cardiovascular risk profile (31).

The Dietary Inflammatory Index-DII (32) was calculated to estimate the overall inflammatory potential of the diet using the standardized methodology described by Shivappa et al. Dietary intake data obtained from the FFQ provided information on 30 of the 45 food parameters included in the original DII. The remaining components were not available from the FFQ and were therefore not included. For each dietary component, individual intake was standardized against a global reference database to generate *z*-scores, which were then converted to centered percentile scores and multiplied by their respective inflammatory effect scores. The overall DII score was calculated by summing these values across all available components, with higher scores indicating a pro-inflammatory dietary pattern.

### 2.3 Perceived pain, functional and wellbeing outcomes

The Short Form-36 Health Survey (SF-36) is an instrument to assess overall quality of life (33) and was previously used in women with endometriosis (34). The SF-36 has demonstrated good psychometric properties in Spanish-speaking populations [Cronbach’s *α* = 0.71–0.94; (35)]. For the purposes of this study were evaluated on a scale of 0 to 100, body pain, vitality and global mental health. A higher score indicates greater involvement of the dimension in quality of life.

The Pain Catastrophizing Scale (PCS) is an instrument that assesses the sensitivity of pain (36). PCS was associated with endometriosis, showing higher levels of disability (37). The PCS has been validated in Spanish populations [Cronbach’s *α* = 0.79; (35)], supporting their reliability and validity. The scale consists of 13 items rated on a Likert scale from 0 to 4, with a total score ranging from 0 to 52. Higher scores indicate greater pain.

The Stellenbosch Endometriosis Quality of Life (SEQOL) assess the specific quality of life in women with endometriosis (38, 39). Although no formal Spanish validation of the SEQOL is currently available, the original instrument has shown high internal consistency (Cronbach’s *α* = 0.92). The version comprises 35 items ranging on a Likert scale from 1 to 5, where higher values indicate greater life functionality. For the propose of this study was evaluated psychological wellbeing, sexual & relationship function, reproductive function, and menstrual characteristics.

### 2.4 Statistical analysis

The analysis was performed by R software version 4.5.2 (R Core Team 2022, Vienna, Austria; https://www.R-project.org/) with the RStudio interface (version 2025.09.2–418 for Windows; Boston, MA, USA). The packages used were *rio, dplyr, compareGroups, tidyverse, ggplot2, ggpubr, rstatix, corrplot, dietaryindex* (40), *caret*, *psych*, and *elasticnet*.

The data was summarized by sample size (n) and relative frequency in categorical variables and median and interquartile range [Q1; Q3] in quantitative variables. Normality of continuous variables was assessed using the visual inspection of Q–Q plots. Between-group comparisons were performed using the Kruskal–Wallis test, followed by Tukey’s Honestly significant differences (HSD) post-hoc test correction for multiple comparisons. In significant pairwise comparisons, effect size was calculated by the rank-biserial correlation (*r*). Effect size was interpreted as small (*r* < 0.30), moderate (*r* = 0.30–0.49), and large (*r* ≥ 0.50). Categorical variables were compared using Fisher’s exact test. The correlations between quantitative variables were tested by Pearson’s *ρ* coefficient, reporting 95% of confidence interval [95% (CI)]. In addition, the adjusted per 1,000 kcal of energy intakes were standardized by mean and standard deviation. Before conducting factor analysis, the suitability of the dataset was assessed using the Kaiser-Meyer-Olkin (KMO) and Bartlett’s test for sphericity. These tests determine if the variables are sufficiently correlated and if the correlation matrix is suitable for dimensionality reduction.

To enhance interpretability and avoid multiple cross-loadings of nutritional variables across components, principal component analysis (PCA) approach was applied, promoting a structure in which each nutrient contributed to the nutritional component (Nut PC). To construct PCA, the number of components was determined based on eigenvalues >1.5, scree plot inspection and interpretability. The proportion of variance explained by each Nut PC was estimated based on the variance of the component scores relative to the total variance of the standardized data. Additionally, considering control group as the reference, linear regression model was constructed separately by each functional and wellbeing outcome, adjusted for significant variables reported in the univariate analysis, exploring the antioxidant and inflammatory pattern association. The beta (*β*) coefficient and the standard error were extracted from the models. No missing data imputation techniques were used in this study. Given the exploratory nature of the study, no formal sample size calculation was performed. The *p*-value (*p*) < 0.05 was considered statistically significant in all analyses.

## 3 Results

### 3.1 Social context and blood parameters

In the cohort, 91.9% was Spanish nationality, having university degree in 89.2% of the cases, and 76.3% of the women were in a relationship. 92.1% were active employees, with income over 4,000€/month in 26.3% of cases. The active smoker was 21.1%. With a statistical trend, lymphocytes were elevated, and cortisol level was significantly lower in women with OMA than control group (moderate effect *r* = 0.49–0.51) (Table 1). The rest of the blood values did not show significant differences between groups.

**Table 1:** Sociodemographic variables and blood parameters by group.

Variables	Control (*n* = 15)	OMA NS (*n* = 17)	OMA S (*n* = 10)	*p*
Age (years)	34.0 [29.0; 36.5]	30.0 [27.0; 37.0]	36.5 [35.8; 41.2]	0.120
Weight (kg)	56.8 [55.0; 59.5]	59.5 [56.0; 68.2]	63.0 [56.2; 69.0]	0.385
Active smoker	2 (13.3%)	2 (15.4%)	4 (50.0%)	0.135
Dietary supplementation	2 (13.3%)	5 (38.5%)	2 (25.0%)	0.326
Allergy	3 (23.1%)	1 (8.33%)	2 (40.0%)	0.287
Platelets (x10^3^/μL)	251 [218; 259]	249 [218; 278]	272 [248; 284]	0.272
Neutrophils (x10^3^/μL)	3.28 [2.63; 4.39]	3.88 [3.12; 5.17]	4.44 [2.79; 6.16]	0.249
Lymphocytes (x10^3^/μL)	1.68 [1.62; 2.02]	2.33 [1.63; 2.53]	2.29 [1.97; 2.46]	0.088
Monocytes (x10^3^/μL)	0.31 [0.28; 0.33]	0.33 [0.25; 0.38]	0.38 [0.26; 0.52]	0.860
Eosinophils (x10^3^/μL)	0.18 [0.12; 0.23]	0.11 [0.06; 0.28]	0.12 [0.08; 0.36]	0.690
Basophils (x10^3^/μL)	0.04 [0.03; 0.05]	0.04 [0.03; 0.04]	0.04 [0.03; 0.06]	0.600
Glucose (mg/dL)	88.0 [81.0; 95.0]	86.0 [83.0; 93.0]	91.5 [87.2; 95.0]	0.485
Cholesterol (mg/dL)	179 [171; 188]	176 [160; 188]	190 [156; 209]	0.851
HDL (mg/dL)	61.0 [52.0; 66.0]	58.0 [53.0; 64.0]	55.0 [49.5; 67.8]	0.680
LDL (mg/dL)	99.0 [93.5; 107]	105 [93.0; 111]	114 [96.2; 128]	0.487
Triglycerides (mg/dL)	66.0 [53.5; 80.5]	67.0 [46.0; 69.0]	64.5 [61.5; 79.0]	0.865
Albumin (mg/dL)	4.50 [4.30; 4.65]	4.60 [4.50; 4.70]	4.50 [4.38; 4.62]	0.429
Creatine (mg/dL)	0.73 [0.68; 0.78]	0.70 [0.65; 0.72]	0.80 [0.66; 0.86]	0.284
Urea (mg/dL)	34.0 [29.0; 38.5]	28.0 [27.0; 34.0]	27.5 [25.0; 32.2]	0.258
Bilirubin (mg/dL)	0.48 [0.40; 0.66]	0.63 [0.49; 1.01]	0.56 [0.46; 0.78]	0.295
Ferritin (ng/dL)	33.0 [23.0; 69.0]	47.0 [18.0; 64.0]	31.0 [15.2; 44.5]	0.771
Cortisol (μg/dL)	18.0 [17.1; 19.9]^a^	9.70 [7.10; 13.2]^b^	10.8 [8.65; 14.2]^b^	0.010
Vitamin D (ng/dL)	20.0 [14.5; 27.0]	30.0 [18.0; 35.0]	23.0 [18.8; 29.2]	0.327
Reactive C-protein (mg/dL)	0.50 [0.50; 2.55]	0.80 [0.50; 1.60]	0.55 [0.50; 1.20]	0.668
Prothrombin (%)	102 [100; 114]	102 [98.0; 112]	108 [103; 116]	0.539
Fibrinogen (mg/dl)	279 [259; 328]	303 [273; 327]	311 [280; 353]	0.734

Data shows median and interquartile range [Q1; Q3]. The *p*-value (*p*) was extracted from Kruskal-Wallis’s test. The groups with different letters indicate *p* < 0.05 by Tukey HSD test. Ovarian endometriosis (OMA) without surgery (NS) or with surgery (S), arbitrary units (a.u.).

### 3.2 Perceived symptoms, functional and wellbeing outcomes

OMA groups exhibited lower scores in psychological wellbeing (large effect *r* = 0.62–0.72), pain sensitivity (large effect *r* = 0.51–0.60), and sexual & relationship function (large effect *r* = 0.51–0.79) than control, indicating a decline in quality-of-life domains. In addition, reproductive function (large effect *r* = 0.63) and menstrual characteristics (large effect *r* = 0.61) was significantly lower in OMA without surgery, but not with surgery, than control. The reproductive function was also lower in OMA without surgery than with surgery, indicating a decline in gynecological and reproductive domains (Table 2).

**Table 2:** Perceived symptoms, functional and wellbeing outcomes by groups.

Functional and wellbeing outcomes	Control (*n* = 15)	OMA NS (*n* = 17)	OMA S (*n* = 10)	*p*
Mental health (a.u.)	21.0 [18.0; 22.5]	17.0 [13.5; 20.5]	17.0 [12.0; 21.0]	0.222
Psychological wellbeing (a.u.)	5.00 [5.00; 5.00]^a^	2.50 [1.85; 2.78]^b^	3.14 [2.71; 3.28]^b^	<0.001
Body pain (a.u.)	18.0 [7.50; 46.0]	42.2 [29.4; 60.8]	41.5 [30.5; 87.5]	0.104
Pain sensitivity (a.u.)	16.0 [13.0; 27.5]^a^	35.5 [27.5; 43.8]^b^	31.0 [25.0; 39.0]^b^	0.003
Vitality (a.u.)	76.9 [53.8; 88.5]	50.0 [32.7; 61.5]	46.2 [30.8; 61.5]	0.051
Sexual & relationship function (a.u.)	5.00 [5.00; 5.00]^a^	3.25 [2.45; 4.30]^b^	4.60 [3.60; 5.00]^b^	<0.001
Reproductive function (a.u.)	5.00 [5.00; 5.00]^a^	2.84 [2.20; 4.64]^b^	4.67 [3.83; 5.00]^a^	0.001
Menstrual characteristics (a.u.)	5.00 [4.66; 5.00]^a^	3.66 [3.08; 4.50]^b^	4.00 [3.66; 5.00]^a,b^	0.005

Data shows median and interquartile range [Q1; Q3]. The groups with different letters indicate *p* < 0.05 by Tukey HSD test. Vitality, body pain and mental health were obtained from the Short Form-36 Health Survey; pain sensitivity was obtained from Pain Catastrophizing Scale; psychological wellbeing, menstrual characteristics, sexual & relationship function and reproductive function were obtained from the Stellenbosch Endometriosis Quality of Life tool. Arbitrary units (a.u.). Ovarian endometriosis (OMA), non-surgery (NS), surgery (S).

In the whole population, lymphocytes did not correlate with the functional and wellbeing outcomes. However, trend towards significance were found between lymphocytes and sexual & relationship function [*ρ* = −0.22 (−0.51; 0.12); *p* = 0.066], and menstrual characteristics [*ρ* = −0.19 (−0.49; 0.15); *p* = 0.051]. Cortisol levels did not show significant correlations with the perceived outcomes, although, again, a trend for a positive correlation with sexual & relationship function [*ρ* = 0.24 [−0.09; 0.53]; *p* = 0.052], and menstrual characteristics [*ρ* = 0.28 (−0.06; 0.55); *p* = 0.098] was observed. The remaining correlations were not statistically significant.

### 3.3 Inflammatory and antioxidant nutritional patterns

The differences in nutritional variables between groups are shown in Supplementary Table S1. The sphericity of the nutritional dataset was adequate (Barlett test: *p* < 0.01; KMO = 0.54). The PCA identified 4 dietary patterns that explained 71% of the overall variance. The complexity of the items was 1.6, the root square of the residuals was 0.07, and the fit based upon off diagonal values was 0.96. In Nut PC1 were clustered mostly antioxidant-products intake (Sum of Squared loadings = 6.07; explained variance = 22%), in Nut PC2 were grouped protein consumption (Sum of Squared loadings = 5.31; explained variance = 20%), in Nut PC3 was related to fat intake (Sum of Squared loadings = 3.72; explained variance = 14%), and Nut PC4 was sugars related to fruits and vegetables (Sum of Squared loadings = 4.12; explained variance = 15%). The rotated load matrix is shown in Supplementary Table S2. All Nut PCs can be interpreted as higher scores indicating greater consumption of the dietary pattern.

**Figure 1:**
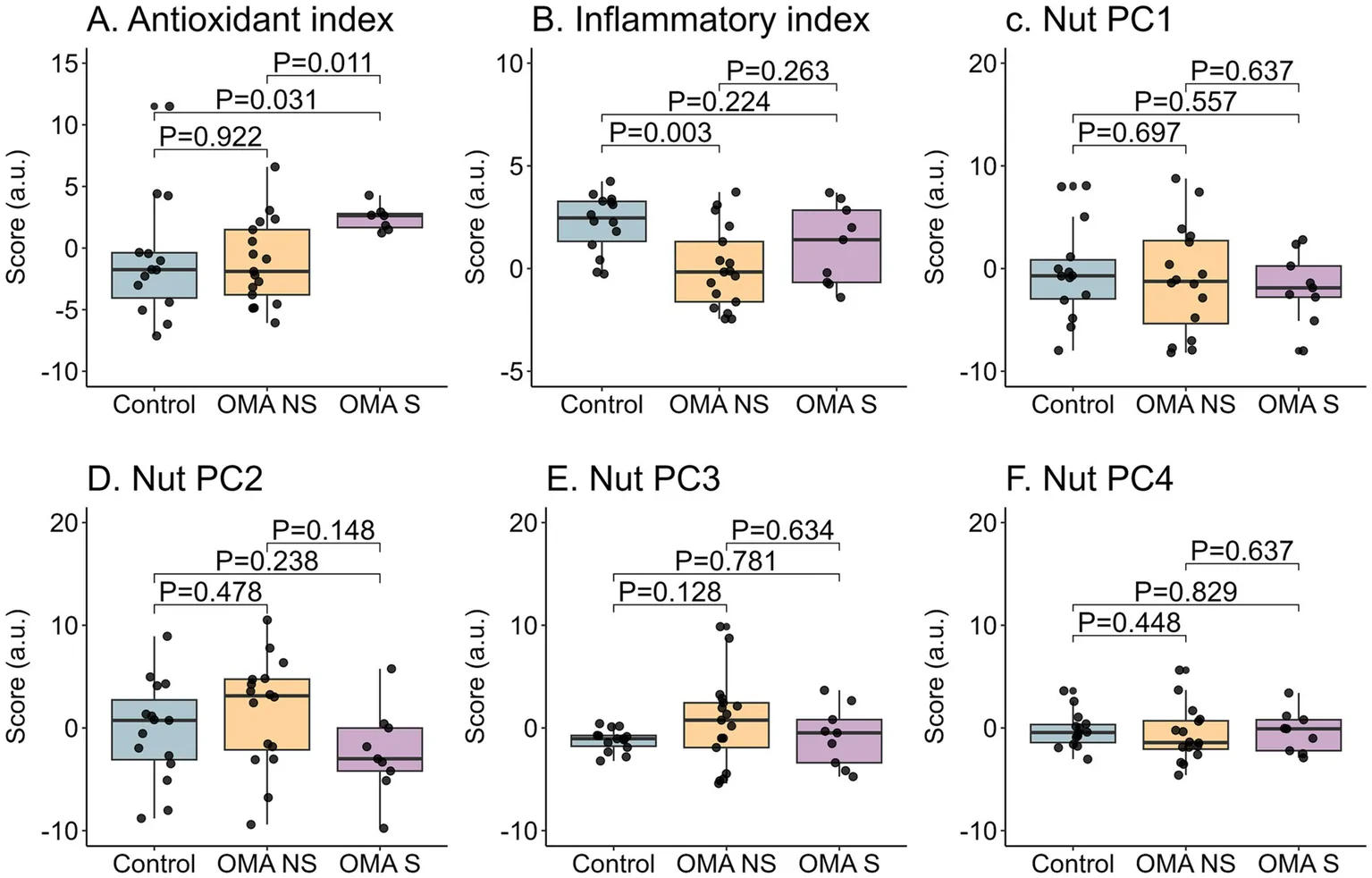
Scores for the principal nutritional components according to group. The data shows median and interquartile range [Q1; Q3]. The *p* value (*p*) was extracted by Tukey HSD test. Arbitrary units (a.u.). Ovarian endometriosis (OMA), non-surgery (NS), surgery (S).

Regarding the dietary index, the DAI was higher among women with OMA with surgery compared to control (moderate effect *r* = 0.47), and OMA without surgery (large effect *r* = 0.51) (Figure 1A). The DII was significantly lower in OMA without surgery than control (large effect *r* = 0.52), and no differences with OMA with surgery (Figure 1B). The Nut PCs scores did not show significant differences between groups (Figures 1C–F).

In the whole population, the DII had negative correlations with Nut PC1. No statistically significant correlations were detected between Nut PCs and DAI scores (Supplementary Table S3). DII and DAI scores were not significantly correlated. Nut PC1 had a positive and significant correlation with Nut PC4 [*ρ* = 0.72 (0.51; 0.84); *p* < 0.001], and Nut PC2 had a negative and significant correlation with Nut PC4 [*ρ* = −0.38 (−0.62; −0.08); *p* = 0.016]. The DII score was positive correlated with cortisol levels [*ρ* = 0.37 (0.04; 0.62); *p* = 0.028]. In addition, Nut PC1 and Nut PC3 were negatively correlated with lymphocytes [Nut PC1: *ρ* = −0.28 (−0.56; −0.00); *p* = 0.050; Nut PC3: *ρ* = −0.48 (−0.70; −0.19); *p* = 0.002].

### 3.4 Inflammatory and antioxidant nutritional patterns and blood parameters

**Figure 2:**
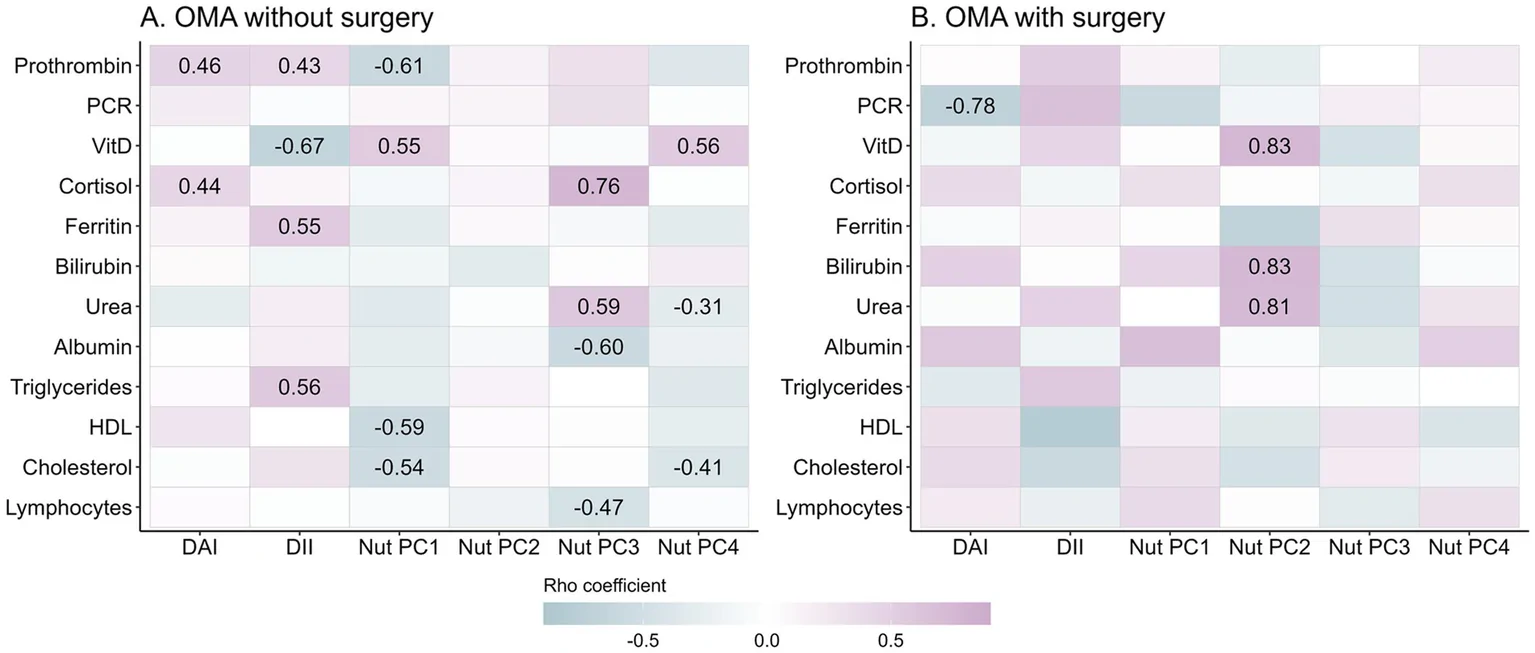
Correlations between nutritional principal components (Nut PC) and blood parameters. The scores were clustered by ovarian endometrioma (OMA) without **(A)** and with surgery **(B)** groups. The cells are colored according to the *rho* coefficient. The significant *rho* coefficient (*p* < 0.05) is shown according to Pearson’s correlation test. Dietary antioxidant index (DAI), dietary inflammatory index (DII), vitamin D (VitD), reactive C-protein (PCR).

In control group, the dietary pattern showed moderate correlations with blood parameters (Supplementary Figure S1). In OMA without surgery, the Nut PC1 pattern showed negative correlations with lipid-related parameters and prothrombin, and positive correlations with vitamin D. In contrast, the Nut PC3 pattern was positively associated with cortisol and urea and inversely correlated to albumin and lymphocyte levels. The Nut PC4 pattern showed negative correlations with cholesterol and urea and positive with vitamin D. Regarding dietary indices, DAI was positively correlated with prothrombin and cortisol, whereas DII showed positive correlations with prothrombin, ferritin and triglycerides and an inverse correlation with vitamin D (Figure 2A). In OMA surgery, the Nut PC2 pattern was positively correlated with vitamin D, bilirubin and urea. Additionally, DAI was inversely correlated with reactive C-protein (Figure 2B).

### 3.5 Association between nutritional patterns and functional and wellbeing outcomes

**Figure 3:**
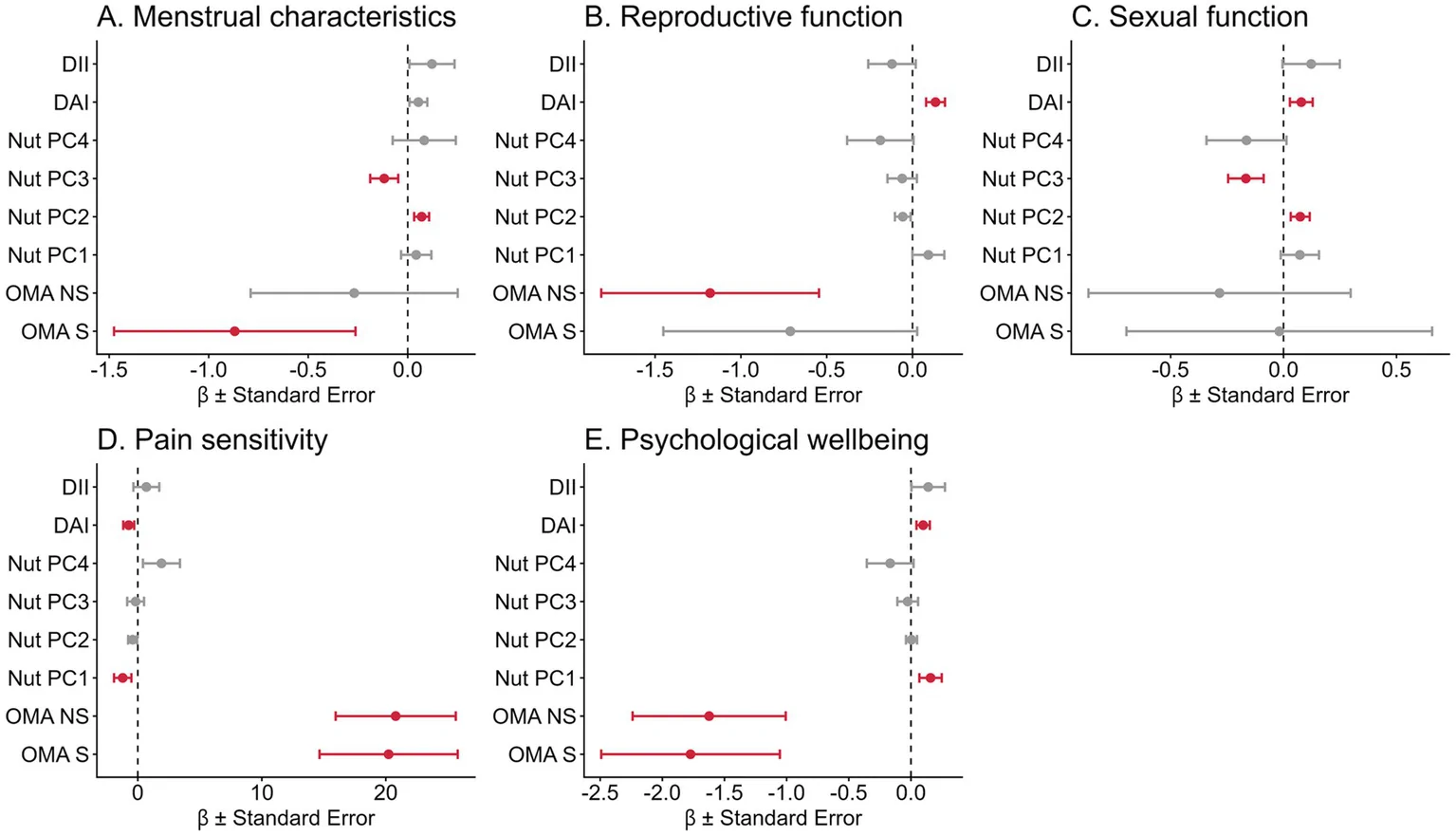
Linear regression models of sparse nutritional principal components (Nut PCs) associate to menstrual characteristics **(A)**, reproductive function **(B)**, sexual function **(C)**, pain sensitivity **(D)**, and psychological wellbeing **(E)**. The data show beta coefficient (*β*) and standard error, being red line a significant association (*p* < 0.05). The reference was the control group, and models were adjusted by lymphocytes and cortisol levels. Pain sensitivity was obtained from Pain Catastrophizing Scale; psychological wellbeing, menstrual characteristics, sexual & relationship function and reproductive function were obtained from the Stellenbosch Endometriosis Quality of Life tool. Ovarian endometrioma without (OMA NS) and with surgery (OMA S), dietary antioxidant index (DAI), dietary inflammatory index (DII).

The adjusted models were performed to examine the associations between dietary and nutritional patterns, functional, and wellbeing outcomes in women with OMA, stratified by non-surgical and surgical treatment. Menstrual characteristics were reduced in women with OMA surgically treated, while Nut PC2 pattern increase and Nut PC3 pattern decrease this score (Figure 3A). Reproductive function was significantly lower in women with OMA without surgical treatment, and antioxidant dietary index increase this score (Figure 3B). Sexual function was reduced with Nut PC3 pattern, and this score increased with high Nut PC2 score and antioxidant dietary index (Figure 3C). Pain sensitivity was higher in women with OMA compared to control. In addition, higher antioxidant dietary index and Nut PC1 pattern were significantly associated with decreased pain sensitivity (Figure 3D). Psychological wellbeing was lower in women with OMA, independently of surgery, and Nut PC1 pattern and antioxidant dietary index increase this outcome (Figure 3E).

## 4 Discussion

Women with OMA display poorer gynecological and psychosocial quality-of-life domains (41). This study provides evidence that antioxidant and inflammatory dietary patterns are differentially associated with functional and wellbeing outcomes in women with OMA undergoing hormonal therapy, with contrasting effects by surgical status. The most striking results can be summarized as follows. The pain sensitivity scores were higher in women with OMA, whereas antioxidant dietary patterns (DAI and Nut PC1) were associated with lower pain score. Conversely, psychological wellbeing increased with these higher dietary scores. Menstrual characteristics scores were reduced in women with surgically treated OMA; however, a nutritional component related to protein intake (Nut PC2) increased these scores, while a lipid-related component (Nut PC3) was associated with their reduction. Reproductive function scores were lower in non-operated OMA, and high DAI were associated with higher scores. Sexual function scores were increased in association with DAI and Nut PC2 patterns, whereas they were decreased in association with Nut PC3. These associations were adjusted by cortisol and lymphocytes, supporting a plausible link between diet, inflammation, and symptoms in OMA.

Endometriosis is a systemic inflammatory disease rather than a local pelvic disorder (1). Our findings showing high lymphocytes levels in women with OMA and their negative correlation with sexual & relational function, and menstrual characteristics underscore the relevance of immune activation in symptom burden. Lymphocytes play a central role in the inflammatory microenvironment of endometriotic lesions (42), releasing reactive oxygen species, proteases, and pro-inflammatory cytokines, thereby amplifying tissue damage and nociceptive signaling (43). Systemic immune activation may bridge processes and patient-reported outcomes, including mental and sexual health. Interestingly, low circulating cortisol levels in women with OMA may reflect hypothalamic–pituitary–adrenal axis dysregulation, which has been described in chronic inflammatory and pain conditions, including endometriosis (44). Given cortisol’s immunomodulatory role, reduced levels may contribute to sustained low-grade inflammation and heightened symptom perception (45).

In this study, a principal component approach was applied to reduce the dimensionality of nutritional variables and to aggregate into components, reflecting the synergistic interactions among nutrients in biological systems. This technique allows us to capture overall dietary behaviors rather than isolated nutrient effects, facilitating the identification of relevant patterns. This strategy has been widely adopted in clinical nutrition and epidemiology to derive dietary constructs associated with health outcomes (46–48). The negative correlations between the dietary inflammatory index (DII) and antioxidant-related nutritional component (Nut PC1) could support the internal validity of the dietary constructs used. In OMA with surgery showed a favorable dietary profile, characterized by high antioxidant, while in OMA without surgery had low inflammatory score, reflecting adaptive dietary behaviors following diagnosis. Similar shifts toward healthier eating patterns have been described in women with endometriosis seeking symptom relief through lifestyle modification (49). However, despite these dietary differences, symptom burden remained high, suggesting that diet alone may not fully counteract the inflammatory milieu associated with established lesions. The observed negative correlation between Nut PC1 and lymphocytes supports the anti-inflammatory potential of plant-based foods rich in polyphenols, fiber, and micronutrients, previously described (50). These compounds have been shown to modulate white blood cells activation, oxidative stress, and cytokine production (51).

One of the most consistent findings of this study is the association between antioxidant dietary patterns and lower pain sensitivity. These results align with interventional and observational studies showing that antioxidant profile, such as Mediterranean diet, reduce pelvic pain and improve quality of life in women with endometriosis (52, 53). Additionally, the present data show that higher fat-related dietary patterns (Nut PC3) were associated with decreased menstrual characteristics and sexual function scores. This finding may be linked to an increased inflammatory milieu, which is consistent with these impairments, which are driven by chronic pelvic inflammation, adhesions, and structural ovarian damage (54). In this context, the upregulation of lipid metabolism observed in pathological states suggests that the accumulation of fatty acids and related metabolites may intensify the inflammatory response, a key factor implicated in the development and progression of endometriosis (55). However, this statement should be taken with caution, since in our data Nut PC3 is a set of nutritional variables that, although it includes total fat intake, also includes unsaturated fatty acids. Specialized pro-resolving mediators are enzymatically derived from polyunsaturated fatty acids, serving as actively promote the resolution of inflammation (56, 57). Thus, although other studies have shown that fat intake could have a detrimental effect on the quality of life of women with endometriosis (5), these data require further investigation. In fact, our models found no direct association between Nut PC3 and pain sensitivity.

Psychological wellbeing was linked to both antioxidant dietary patterns, highlighting the interplay between diet and mental health. In chronic inflammation environment, pro-inflammatory cytokines, such as IL-1β and TNF-α, change neurotransmitter metabolism and disrupt neural plasticity (58, 59). Emerging evidence indicates that dietary patterns modulate mood like antioxidants diets and fiber are associated with improved psychological outcomes, reducing inflammatory profiles, whereas pro-inflammatory dietary patterns are linked to poorer mental health in adults (60, 61). Women with endometriosis frequently correlate with systemic pro-inflammatory markers (2, 62) and dietary modulation has been proposed as a strategy to attenuate inflammation by oxidative stress and symptom severity, which may in turn influence quality of life and psychological wellbeing (49, 63).

Menstrual, sexual and reproductive function emerged as domains particularly sensitive to both dietary patterns and surgical status. Women with non-surgical OMA exhibited poorer reproductive and sexual function, consistent with the mechanical, inflammatory, and neuroendocrine effects of persistent ovarian lesions (64, 65). In this group, higher protein-related intake was positively associated with menstrual characteristics and sexual function. This could support lifestyle factors, such as diet, and can have measurable effects on symptom perception.

Taken together, these findings suggest that diet may act as a modifier rather than a primary determinant of symptoms in OMA. Antioxidant-rich dietary patterns appear to support immune modulation and symptom relief, whereas pro-inflammatory patterns may exacerbate pain and sexual dysfunction. Lymphocytes and cortisol emerge as biological link between diet and symptomatology, reinforcing their potential role as accessible biomarkers in nutritional and lifestyle interventions.

### 4.1 Limitations, clinical implications and future perspectives

Several limitations should be acknowledged. First, the cross-sectional design precludes causal inference regarding temporal relationships between diet, inflammation, and symptoms. Women with severe symptoms may have modified their dietary habits, through professional nutritional counseling, which may have influenced the observed associations. In addition, dietary intake was self-reported, which may introduce recall bias despite validated assessment tools. For both reasons, it would be desirable to perform longitudinal studies. Secondly, all women were under hormonal therapy, which may have modulated inflammatory markers and symptom perception, potentially attenuating dietary associations. Moreover, information regarding the use of analgesics was not systematically collected, and this factor may also have influenced symptom severity. Furthermore, although women in the control group had no clinical diagnosis, the absence of laparoscopic confirmation means that asymptomatic endometriosis cannot be completely excluded. Finally, it would be interesting to determine other confounding factors, such as physical activity (66), stress (67), and sleep quality (68) that may influence these relationships.

Future research should prioritize interventional designs to clarify causality and explore whether dietary modification can meaningfully reduce symptom burden in OMA. Mediation analyses using structural equation modeling could further elucidate whether systemic inflammatory markers mediate the relationship between dietary patterns and clinical outcomes. Recent evidence of dietary interventions in endometriosis suggests that anti-inflammatory, Mediterranean, low-FODMAP, and antioxidant-rich diets may improve pain and quality of life (69). Additionally, although emerging RCTs suggest that dietary interventions may alleviate specific symptoms in endometriosis, evidence remains scarce and methodologically heterogeneous. Thus, RCTs comparing anti-inflammatory dietary interventions in surgically versus non-surgically treated women are warranted to determine whether surgical status modifies dietary responsiveness.

From a clinical perspective, these findings support the integration of nutritional counseling into the multidisciplinary management of OMA. While diet should not replace surgical or hormonal treatment, promoting antioxidant-rich and anti-inflammatory dietary patterns may offer a low-risk adjunctive strategy to improve pain perception, psychological wellbeing, and reproductive-related quality of life, particularly in women with persistent disease. Personalized dietary approaches that consider disease stage, surgical history, and systemic inflammatory status may represent a promising avenue for optimizing care in endometriosis.

## 5 Conclusion

Nutrition may play a meaningful, although not exclusive, role in shaping how symptoms are experienced by women with ovarian endometriosis receiving hormonal treatment. Dietary patterns characterized by higher antioxidant intake were associated with lower pain sensitivity and better psychological wellbeing, while fat-dominant patterns tended to align with poorer menstrual characteristics and sexual function. These associations were dependent, at least in part, on surgical history, highlighting that the biological environment created by lesion persistence may condition the extent to which diet can influence symptom perception. The links between dietary patterns, circulating lymphocytes, and patient-reported outcomes reinforce the idea that systemic inflammation remains a key pathway connecting lifestyle factors with the lived experience of endometriosis. Rather than acting as a standalone solution, diet seems to function as a modulator of an already complex inflammatory and hormonal landscape. These results support an integrative view of endometriosis management, in which nutritional habits are considered alongside surgical and pharmacological strategies. Encouraging antioxidant-rich, anti-inflammatory dietary patterns may offer a feasible and patient-centered way to complement conventional treatments, with the potential to improve quality of life in women with ovarian endometrioma.

## Data Availability

The raw data supporting the conclusions of this article will be made available by the authors, without undue reservation. Further inquiries can be directed to the corresponding author/s.
