# Supplementary material for: Antioxidant dietary patterns mitigate pain and raise psychological wellbeing in ovarian endometrioma: surgical status shapes associations
Source: Front Nutr. 2026 Aug 12;13:1921186. doi: 10.3389/fnut.2026.1921186 (PMC13506264; doi:10.3389/fnut.2026.1921186)
Supplement: Supplementary file 1 [file Table_1.DOCX]

Supplementary Material

# 1 Supplementary Data

**Table S1.** Nutritional elements by groups.

| **Nutritional elements** | **Control (n=15)** | **OMA NS (n=17)** | **OMA S (n=10)** | **P** |
| --- | --- | --- | --- | --- |
| Carbohydrates (g*1000kcal/day) | 112 [100; 123] | 105 [93.7; 121] | 118 [114; 125] | 0.259 |
| Total sugar (g*1000kcal/day) | 62.4 [54.6; 67.2] | 51.5 [44.8; 60.2] | 64.9 [47.7; 66.0] | 0.162 |
| Sucrose (g*1000kcal/day) | 21.1 [17.8; 22.8] | 20.3 [17.2; 22.4] | 24.9 [22.7; 29.5] | 0.083 |
| Proteins (g*1000kcal/day) | 51.5 [49.4; 61.8] | 53.0 [45.7; 59.8] | 48.2 [43.6; 54.4] | 0.362 |
| Fats (g*1000kcal/day) | 37.5 [35.3; 40.3] | 42.0 [32.7; 45.9] | 40.9 [35.9; 42.9] | 0.423 |
| Cholesterol (mg*1000kcal/day) | 150 [118; 169] | 171 [137; 214] | 147 [124; 173] | 0.321 |
| SFAs (g*1000kcal/day) | 12.5 [11.9; 14.3] | 13.1 [11.8; 14.5] | 13.6 [10.4; 14.9] | 0.954 |
| MUFAs (g*1000kcal/day) | 15.4 [13.6; 15.9] | 16.9 [12.1; 20.0] | 17.9 [14.3; 19.3] | 0.283 |
| PUFAs (g*1000kcal/day) | 6.75 [5.35; 7.49] | 6.90 [6.17; 8.65] | 6.61 [5.76; 7.39] | 0.521 |
| Vitamin A (μg*1000 kcal/day) | 465 [364; 568] | 484 [369; 733] | 578 [512; 630] | 0.397 |
| Vitamin C (mg*1000 kcal/day) | 71.2 [46.7; 82.1] | 64.6 [40.8; 99.1] | 54.3 [36.5; 60.2] | 0.259 |
| Vitamin D (μg*1000 kcal/day) | 1.64 [1.14; 1.91] | 1.72 [1.42; 2.29] | 1.51 [1.12; 1.61] | 0.575 |
| Vitamin E (mg*1000 kcal/day) | 6.23 [5.47; 6.85] | 6.56 [5.94; 7.00] | 5.66 [5.15; 6.53] | 0.315 |
| β-carotene (μg*1000 kcal/day) | 1745 [984; 2159] | 1778 [871; 2416] | 1700 [1008; 1819] | 0.825 |
| Total carotene (μg*1000 kcal/day) | 1916 [1079; 2402] | 1920 [1003; 2751] | 1963 [1090; 2082] | 0.880 |
| Total folate (μg*1000 kcal/day) | 145 [135; 176] | 136 [109; 145] | 130 [118; 132] | 0.150 |
| Total fiber (g*1000 kcal/day) | 8.39 [7.60; 9.70] | 9.05 [6.13; 10.3] | 8.15 [7.40; 9.17] | 0.818 |
| Selenium (μg*1000 kcal/day) | 36.7 [31.2; 42.3] | 41.4 [32.2; 46.4] | 37.8 [30.8; 48.0] | 0.636 |
| Zinc (mg*1000 kcal/day) | 5.93 [5.53; 6.48] | 6.39 [4.86; 6.72] | 5.12 [4.71; 6.39] | 0.285 |
| Magnesium (mg*1000 kcal/day) | 194 [182; 206] | 194 [160; 202] | 150 [138; 186] | 0.116 |
| Iron (mg*1000 kcal/day) | 5.87 [5.57; 6.01] | 5.98 [5.04; 6.37] | 5.68 [5.59; 5.93] | 0.888 |
| Sodium (mg*1000 kcal/day) | 1269 [1100; 1381] | 1429 [1269; 1616] | 1309 [1171; 1711] | 0.196 |
| Fruits products (g*1000kcal/day) | 180 [164; 216] | 133 [104; 252] | 78.3 [59.7; 145] | 0.240 |
| Vegetable products (g*1000kcal/day) | 156 [79.3; 214] | 136 [64.8; 201] | 133 [106; 154] | 0.673 |
| Fish products (g*1000kcal/day) | 31.0 [16.2; 49.7] | 27.1 [16.0; 43.2] | 19.1 [18.2; 28.1] | 0.756 |
| Meat products (g*1000kcal/day) | 77.6 [39.6; 84.5] | 78.0 [45.0; 110] | 61.7 [58.3; 79.4] | 0.471 |
| Snacks products g*1000kcal/day) | 13.0 [6.95; 15.9] | 16.3 [7.87; 22.1] | 15.9 [8.91; 18.6] | 0.421 |

Data shows median and interquartile range [Q1; Q3]. All nutritional elements were standardized per 1000 kcal/day of energy intake. Ovarian endometriosis (OMA); non-surgery (NS); surgery (S); saturated fatty acids (SFAs); monounsaturated fatty acids (MUFAs,); polyunsaturated fatty acids (PUFAs).

**Table S2.** Rotated matrix load factors and nutritional principal components (Nut PC).

| **Nutrient elements** | **Nut PC1** | **Nut PC2** | **Nut PC3** | **Nut PC4** | **h^2^** | **u^2^** |
| --- | --- | --- | --- | --- | --- | --- |
| Vegetables products | 0.887 |  |  |  | 0.847 | 0.153 |
| β-carotenes | 0.886 |  |  |  | 0.813 | 0.187 |
| Total carotenes | 0.873 |  |  |  | 0.786 | 0.214 |
| Total folates | 0.833 |  |  |  | 0.831 | 0.169 |
| Iron | 0.806 |  |  |  | 0.729 | 0.271 |
| Vitamin A | 0.767 |  |  |  | 0.672 | 0.328 |
| Total fiber | 0.653 |  |  | 0.611 | 0.902 | 0.098 |
| Vitamin C | 0.622 |  |  | 0.629 | 0.801 | 0.199 |
| Magnesium | 0.610 |  |  |  | 0.591 | 0.409 |
| Proteins |  | 0.892 |  |  | 0.875 | 0.125 |
| Selenium |  | 0.858 |  |  | 0.765 | 0.235 |
| Cholesterol |  | 0.756 |  |  | 0.708 | 0.292 |
| Vitamin D |  | 0.714 |  |  | 0.576 | 0.424 |
| Fish products |  | 0.711 |  |  | 0.684 | 0.316 |
| Zinc |  | 0.711 |  |  | 0.726 | 0.274 |
| Meat products |  | 0.584 |  |  | 0.594 | 0.406 |
| Snack products |  | -0.517 |  |  | 0.460 | 0.540 |
| Sucrose |  | -0.528 |  | 0.563 | 0.607 | 0.393 |
| Carbohydrates |  | -0.570 | -0.668 |  | 0.911 | 0.089 |
| Fats |  |  | 0.920 |  | 0.948 | 0.052 |
| MUFAs |  |  | 0.848 |  | 0.727 | 0.273 |
| PUFAs |  |  | 0.784 |  | 0.634 | 0.366 |
| Vitamin E |  |  | 0.579 |  | 0.773 | 0.227 |
| Sodium |  |  | -0.483 |  | 0.288 | 0.712 |
| Fruits products |  |  |  | 0.819 | 0.775 | 0.225 |
| Total sugar |  |  |  | 0.668 | 0.678 | 0.322 |
| SFAs |  |  |  | -0.631 | 0.525 | 0.475 |
| SS loadings | 6.07 | 5.31 | 3.72 | 4.12 |  |  |
| Explained variance | 0.22 | 0.20 | 0.14 | 0.15 |  |  |

Saturated fatty acids (SFAs); monounsaturated fatty acids (MUFAs,); polyunsaturated fatty acids (PUFAs); communality (h^2^); uniquenesses (u^2^), Sum of Squared (SS).

**Table S3.** Correlations between inflammatory and antioxidant dietary indices and nutritional principal components (Nut PC).

|  | **Dietary antioxidant index** | **Dietary inflammatory index** |
| --- | --- | --- |
| **Nut PC1** | ρ=-0.01 [-0.33; 0.32]; P=0.967 | ρ=-0.44 [-0.66; -0.14]; P=0.006 |
| **Nut PC2** | ρ=0.06 [-0.27; 0.37]; P=0.738 | ρ=0.03 [-0.28; 0.34]; P=0.834 |
| **Nut PC3** | ρ=-0.12 [-0.42; 0.22]; P=0.497 | ρ=-0.16 [-0.45; 0.17]; P=0.336 |
| **Nut PC4** | ρ=0.08 [-0.25; 0.40]; P=0.632 | ρ=-0.21 [-0.49; 0.11]; P=0.201 |

The data shows Rho (ρ) coefficient and the 95% confidence interval. The P-value (P) was extracted from Pearson's correlation test.

**Figure S1**. Correlations between nutritional principal components (Nut PC) and blood parameters in control group. The cells are colored according to the Rho coefficient. The significant rho coefficient (P<0.05) is shown according to Pearson's correlation test. Dietary antioxidant index (DAI), dietary inflammatory index (DII), Vitamin D (VitD), Reactive C-protein (PCR).
